# Effect of Neural Therapy on shoulder dysfunction and pain in supraspinatus tendinopathy

**DOI:** 10.12669/pjms.38.3.4823

**Published:** 2022

**Authors:** Ibrahim Bashan, Gulsah Yasa Ozturk

**Affiliations:** 1 Ibrahim Bashan, MD. Medical Education Department, Mersin University, Faculty of Medicine, Mersin, Turkey; 2 Gulsah Yasa Ozturk, MD. Physical Medicine and Rehabilitation Department, University of Health Sciences, Adana City Hospital Adana, Turkey

**Keywords:** Neural Therapy, Procaine, Musculus Supraspinatus Tendinopathy

## Abstract

**Objectives:**

To investigate the effect of 1% procaine injection, which is used in neural therapy, on shoulder pain and dysfunction in patients diagnosed with supraspinatus tendinopathy.

**Methods:**

The Range of Motion values, Visual Analog Scale and the QuickDASH Scale scores of 70 patients, who were diagnosed with musculus supraspinatus tendinitis based on magnetic resonance imaging findings, were analyzed. The data of the scales obtained before neural therapy and at the follow-up visit at four weeks after the end of therapy were compared, and a p-value of <0.05 was considered statistically significant.

**Results:**

After neural therapy, a statistically significant increase was observed in Range of Motion values and there was a statistically significant decrease in both the Visual Analog Scale and QuickDASH score averages.

**Conclusion:**

This is one of the rare studies showing the effects of neural therapy application on shoulder pain severity and dysfunction in patients with supraspinatus tendinitis who are resistant to medical therapy.

## INTRODUCTION

Shoulder impingement syndrome (SIS) is the most common cause of shoulder pain and dysfunction and typically results from the compression of the supraspinatus muscle tendon below the acromion.^1^,^2^ The initial treatment of SIS is conservative and aims to improve pain and inflammation with non-steroidal anti-inflammatory drugs and controlled physical therapy regimens.^3^ If symptoms persist, injections of subacromial local anesthetics and corticosteroids may be indicated to relieve pain.^4^ However, despite their frequent use, a meta-analysis of randomized clinical trials evaluating subacromial corticosteroid injection showed that these injections might have limited short-term benefit.^5^ Successful results have been reported regarding the effectiveness of neural therapy (NT) with 1% procaine or lidocaine injection in treatment-resistant musculoskeletal diseases.^6^ It is suggested that local anesthetics achieve this effect by triggering parasympathetic stimuli and disrupting the vicious cycle in pain.^7^

NT is a simple and effective method of treatment by injecting local anesthetics into the most commonly symptomatic trigger points or alternatively into autonomic ganglia, scars, and other tissues.^8^ Although theoretically, parasympathetic stimulation is known to be effective in the mechanism of NT, the results regarding the experimental proof are unclear.^9^ Procaine, one of the first-choice local anesthetic agents in NT, is applied at a rate of 0.5-1%, and para-amino benzoic acid and diethylaminoethanol, which are metabolites of procaine, contribute to the regulation of the cell by regulating endothelial function. In addition, short duration of action and absence of sympathomimetic substances are among the most important reasons for the preference of procaine.^10^ In recent years, the importance of NT has increased, and its application area has spread from Central Europe to other countries.^11^,^12^ Thus, this study aimed to investigate the possible positive effect of NT on shoulder pain and dysfunction.

## METHODS

### Study design and data collection

The study population consisted of patients who presented to the physical therapy and rehabilitation outpatient clinic of a tertiary treatment center with the complaint of shoulder pain in February 2020 and March 2021. After obtaining anamnesis and performing a physical examination, shoulder magnetic resonance imaging (s-MRI) was undertaken, and the patients with findings compatible with supraspinatus tendinitis were retrospectively examined. Seventy patients who were resistant to medical treatment (previously used local, intramuscular or oral analgesic, anti-inflammatory and antimuscarinic agents), had neck pain for more than three months, and accepted neural therapy, were included in the sample.

In this study, the range of motion (ROM) measurement technique was based on the guidelines of Kendall et al. In the supine position, shoulder flexion, abduction, internal rotation, external rotation, adduction; In the prone position, shoulder extension ROM measurements were made actively, each measurement was repeated three times and their average values were recorded^13^. In addition, the Visual Analog Scale (VAS) was used for the evaluation of shoulder pain and the QuickDASH Scale (Q-DASH) for the evaluation of shoulder dysfunction.^14^,^15^ Data obtained at the initial visit and at four weeks after treatment were compared.

### The exclusion criteria

Patients under the age of 18 years, pregnant women, patients with a history of who surgery in the shoulder girdle area, and those with inflammatory diseases or a history of cancer were excluded from the study.

### Treatment protocol

For all the patients, a total of three sessions of NT was performed at one-week intervals by a physical therapy specialist with a certificate of NT. In the first session, 1% procaine injection was administered to form a lentil-sized papule into the skin at painful points found by palpation around the shoulder. In addition, Quaddel was applied, and 0.5 cc 1% procaine was deep injected into segmental trigger points (into fibrositic nodules) detected by palpation. In the second session, the treatment protocol applied in the first session was repeated, and 1% procaine was injected to form a lentil-sized papule into the skin of the C5-T1 segments, which are the shoulder innervation segments. In the last session, the protocol used in the second session was repeated.^14^,^16^

This study was approved by the local ethics committee of the training and research hospital (approval date: 03.10.2021, number: 1321-76).

### Statistical Analysis

Data obtained and additional s-MRI results were analyzed using SPSS v. 21 software package. The normality of continuous variables was evaluated using the Shapiro-Wilk test. The paired t-test was used for the comparison of variables before and after treatment for normally distributed data, and the Wilcoxon test was conducted for those that did not conform to a normal distribution. Descriptive statistics were given as mean or median values with 95% confidence intervals. Statistical significance was accepted as p < 0.05. Spearman’s Rho correlation coefficient was used to determine the linear relationship between relative changes.

## RESULTS

The demographic distributions of the patients are shown in Table-I. Of the patients, 40 (57.1%) were women and 30 (42.9%) were men.A statistically significant increase was observed in ROM values evaluating shoulder joint range of motion after NT in all movements (extension, flexion, abduction, adduction, internal and external rotation) compared to the baseline data (p < 0.001).

**Table-I:** Demographic characteristics of the patients.

	Mean ± SD	Median (IQR)	Min-Max
Age (year)	51.78 ± 10.47	51.5 (44-59.25)	20-74
Weight (kg)	73.32 ± 10.22	74.0 (65-80)	54-97
Height (cm)	165.0 ± 8.65	164.0 (159.5-170)	150-192
BMI (kg/m^2^)	26.98 ± 3.67	26.55 (24.45-29.30)	19.49-36.79

SD: standard deviation, IQR: interquartile range, BMI: body mass index.

While an average decrease of 73.6% was observed in the VAS score after treatment compared to pre-treatment, this rate was observed as 59.95% for the Q-DASH score. After NT, the VAS scores evaluating shoulder pain and the Q-DASH scores indicating shoulder dysfunction were observed to have statistically significantly decreased compared to the baseline data (p < 0.001). Table-II & III

**Table-II:** Comparison of the baseline and post-NT (after four weeks) ROM scores.

ROM Flexion	Mean ± SD	Median (IQR)	Min-Max	95% CI for Mean	
Baseline	146.00±21.61	155 (140-160)	90-170	140.85-151.15	p< 0.001 effect size: 0.680
Post NT	172.36±7.01	175 (170-180)	150-180	170.69-174.03	
Relative change to baseline (%)	20.79±19.36	12.7 (9.09-22.7)	2.94-78.95	16.17-25.41	
***ROM Extension***					
Baseline	31.57±7.50	35 (25-40)	15-45	29.78-33.36	p < 0.001 effect size: 0.633
Post NT	41.07±3.50	40 (40-45)	35-45	40.24-41.91	
Relative change to baseline (%)	38.38±40.37	28.57 (12.5-60)	-12.5-200	28.75-48.00	
***ROM Abduction***					
Baseline	146.29±22.53	155 (140-161.25)	80-170	140.91-151.66	p < 0.001 effect size: 0.624
Post NT	172.07±5.93	170 (170-175)	150-180	170.66-173.48	
Relative change to baseline (%)	21.04±23.13	12.9 (6.2-25)	0-106.25	15.52-26.55	
***ROM Adduction***					
Baseline	30.64±7.47	30 (25-35)	5-40	28.86-32.42	p < 0.001 effect size: 0.703
Post NT	41.29±3.58	40 (40-45)	35-45	40.43-42.14	
Relative change to baseline (%)	49.26±86.17	33.33 (14.29-60)	-12.5-700	28.71-69.8	
***ROM Internal Rotation***					
Baseline	67.57±10.21	70 (60-75)	45-85	65.14-70.00	p < 0.001 effect size: 0.768
Post NT	83.50±5.54	85 (80-90)	70-90	82.18-84.82	
Relative change to baseline (%)	26.00±18.06	21.43 (12.5-38.46)	0-77.78	21.70-30.31	
***ROM External Rotation***					
Baseline	68.43±9.65	70 (60-75)	40-80	66.13-70.73	p < 0.001 effect size: 0.704
Post NT	83.36±5.09	85 (80-86.25)	75-90	82.14-84.57	
Relative change to baseline (%)	24.30±19.37	23.08 (11.16-34.09)	-6.25-87.5	19.69-28.92	

p: Wilcoxon test, IQR: interquartile range, ROM: range of motion, SD: standard deviation, NT: neural therapy, CI: confidence interval.

**Table-III:** Comparison of the baseline and post-NT (after four weeks) VAS and Q-DASH scores.

	Mean ± SD	Median (IQR)	Min-Max	95% CI for Mean	
Baseline VAS	8.14 ± 1.11	8 (7-9)	6-10	7.88-8.41	p< 0.001 effect size: 0.970
Post-NT VAS	2.20 ± 1.2	2 (1-3)	0-5	1.91-2.49	
Relative change to baseline VAS (%)	73.62 ± 13.11	75 (65.63-85.71)	42.86-100	70.50-76.75	
Baseline Q-Dash	74.55 ± 20.12	75 (61.3-89.18)	31.8-125	69.75-79.35	p < 0.001 effect size: 0.855
Post-NT Q-DASH	31.07 ± 18.95	25 (20.4-50)	0-79.5	26.55-35.59	
Relative change to baseline Q-DASH (%)	59.95±22.04	64.21 (41.86-74.39)	11.92-100	54.69-65.20	

p: Wilcoxon test, IQR: interquartile range, VAS: Visual Analog Scale, SD: standard deviation, NT: neural therapy, CI: confidence interval, Q-DASH: QuickDASH.

A moderate, positive, significant relationship was found between the rate of decrease in the VAS score and the Q-DASH score after treatment compared to the pre-treatment evaluation (r = 0.438; p < 0.001) (p: Spearman’s Rho, (Relative change = |Baseline- PostNT| /Baseline*100).

## DISCUSSION

This is one of the most comprehensive studies showing the effects of NT application on shoulder pain severity, dysfunction and ROM in patients with supraspinatus tendinitis, who have chronic shoulder pain resistant to medical treatment and have not decided to undergo surgery.

Complementary medicine approaches have found a place in the management of patients with treatment-resistant SIS. In a randomized controlled study investigating the effectiveness of acupuncture, after acupuncture treatment, functional improvements were achieved compared to the control group, and pain scores decreased.^17^ In another study, the efficacy of steroid and acupuncture treatments was compared, and no significant difference was found between the two groups in terms of pain and functionality.^18^ In addition, in a systematic review evaluating the effectiveness of injection treatments in patients with rotator cuff lesions, it was mentioned that the effect of even frequently used corticosteroid injections on range of motion, pain and functionality was controversial.^19^

Although there are studies in the literature reporting successful results with the use of NT in chronic musculoskeletal pain, research on its use in SIS is rare. In a patient who was followed up with a diagnosis of frozen shoulder, improvement was found in both pain scores and range of motion measurements of the patient with the application of NT.^20^ Similarly, in a study of 12 patients in which neural therapy efficacy and intraarticular corticosteroid injection were compared in rotator cuff-related shoulder pain, significant improvement was found in the VAS scores and range of motion compared to the corticosteroid group after one month of treatment.^21^ Similar to our study, NT was applied to patients with shoulder pain for more than six months, and a statistically significant decrease was found in the VAS and Q-DASH scores when the baseline and post-treatment first-month data were compared. Unlike our study, the researchers evaluated 17 patients using lidocaine.^22^ In the current study, there was a significant improvement in the post-treatment ROM, VAS and Q-DASH scores indicating pain and functional status, respectively, and we also determined a positive correlation between the pre- and post-treatment differences in the score percentages of VAS and Q-DASH scales, which could reduce handicaps concerning subjective measurement tools.

### Limitations of the study

The main limitations of this study are the small number of patients and the lack of output regarding the long-term results of NT.

## CONCLUSION

NT involves much more than local injections, and it also includes other segmental and trigger point injections that should be evaluated from a holistic perspective. This study showed that NT might be an effective treatment method in patients with shoulder pain and functional limitation due to supraspinatus tendinitis.

### Authors’ Contribution:

**GYO:** Conceived the idea, designed the study, and performed data collection and editing of manuscript.

**IB:** Prepared the manuscript, performed statistical analysis and responsible for integrity of the study.
